# A Case Report on Acute Visual Loss With Ophthalmoplegia Following Spine Surgery

**DOI:** 10.7759/cureus.68453

**Published:** 2024-09-02

**Authors:** Abinaya Ramakrishnan, Siddhartha Singh, Sanjeev K Puri

**Affiliations:** 1 Department of Ophthalmology, Saveetha Medical College and Hospitals, Saveetha Institute of Medical and Technical Sciences, Saveetha University, Chennai, IND

**Keywords:** nonocular surgery, ophthalmic artery occlusion, spine surgery complications, ophthalmoplegia, sudden vision loss

## Abstract

Postoperative vision loss is an unusual but serious side effect that can occur after nonocular surgeries, particularly those involving the heart or spine. Various causes, including ischemic optic neuropathy, central retinal artery occlusion, central retinal vein occlusion, and ischemic orbital compartment syndrome, can cause this condition. Here, we present a case of a 28-year-old male patient who underwent spine surgery for cervicodorsal spine injury and experienced sudden, painless vision loss in his left eye following the surgery. On examination, his right eye had a bedside vision of >3/60, while his left eye could only perceive light. The patient's left eye showed mild axial proptosis, supraorbital edema, conjunctival congestion, chemosis, relative afferent papillary defect, and restricted eye movements in all gazes. Fundus examination of the left eye showed pale retina, optic disc pallor, severely attenuated retinal vessels, and an absent cherry red spot suggesting ophthalmic artery occlusion. The right eye anterior segment and fundus findings were normal. Magnetic resonance imaging of the brain and orbit showed mild preseptal thickening in the left orbit, and magnetic resonance venography was normal. This case report is noteworthy in that an ophthalmic artery occlusion has been identified as the cause of sudden, unilateral, painless vision loss associated with ophthalmoplegia subsequent to a spinal surgical procedure.

## Introduction

Postoperative visual loss (POVL) following nonocular surgery has been gaining attention due to the increasing number of reported cases and estimated rates between 0.05% and 1.0% [1]. It is usually common following cardiac, spine, and head and neck surgeries. The rates of its occurrence in heart and spine surgeries are estimated to be as high as 4.5% and 0.2%, respectively [2,3]. POVL is an unusual but serious complication that poses significant challenges to patients and healthcare providers [3]. Although spinal surgeries are generally considered safe and are commonly performed to address various spinal pathologies, the occurrence of postoperative visual impairment, particularly acute onset, can dramatically alter the patient's quality of life [4]. Several causes, such as ischemic optic neuropathy (ION), central retinal artery occlusion (CRAO), central retinal vein occlusion, and cortical blindness, have been identified in these cases [5].

Preoperative and intraoperative risk factors that may contribute to POVL include systemic comorbidities like diabetes and hypertension, prone position, smoking, renal failure, hemorrhagic shock, intraoperative massive blood loss, anemia, hypothermia, coagulopathy disorders, direct trauma, embolism, and prolonged compression to the eye [1]. Embolic events that occlude arterial lumens around the visual system can cause sudden visual loss due to the occlusion of the retinal arteries [6]. An occluded retinal artery will eventually open and reperfuse the retina over time, but the visual recovery is usually poor [7]. Sometimes, carotid artery dissection, lesions at the orbital apex, or pituitary apoplexy might be the causes of POVL, especially if associated with ophthalmoplegia [8].

Rarely, ophthalmic artery occlusion has been attributed to POVL following nonocular surgery. Hence, this case report is noteworthy in that an ophthalmic artery occlusion has been identified as the cause of sudden, unilateral, painless loss of vision associated with ophthalmoplegia following spine surgery. Visual loss in conjunction with ophthalmoplegia following a nonocular surgery is seldom observed. This report adds to the expanding body of literature on postoperative complications, emphasizing the need for heightened awareness and proactive measures in the surgical and postoperative care of patients undergoing spine surgery.

## Case presentation

A 28-year-old male presented to our hospital with C7-D1 traumatic subluxation with spinal cord injury following a fall from height. The patient underwent C7-D1 decompressive laminectomy with C7-D1 discectomy and stabilization in the prone position for around 5 hours with the head fixed in a horseshoe. The preoperative hemoglobin level was 12.7 g/dL. The surgery was uneventful, with a blood loss of around 200 mL, and the vitals were within normal limits preoperatively as well as intraoperatively. Postoperatively, once the patient gained consciousness, he immediately complained of painless vision loss in the left eye, for which an ophthalmology opinion was sought. Due to the postsurgical and nonambulatory status of the patient, the visual status was assessed at the bedside. It revealed right eye bedside vision of >3/60 and only perception of light in the left eye.

**Figure 1:**
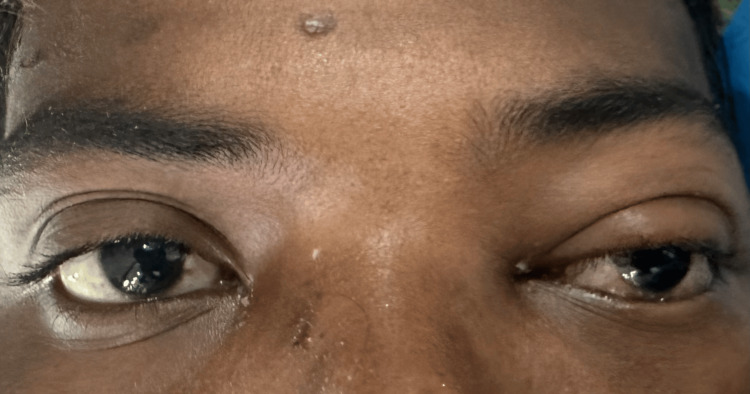
Supraorbital edema, conjunctival congestion, and chemosis in the left eye

Upon assessment, the left eye displayed mild axial proptosis and supraorbital edema. Additionally, the conjunctiva showed signs of mild congestion and chemosis, as shown in Figure 1. On pupillary examination, a relative afferent papillary defect was noted. In addition, restriction of extraocular movements in all gazes was seen. The right eye's anterior segment findings were found to be normal.

**Figure 2:**
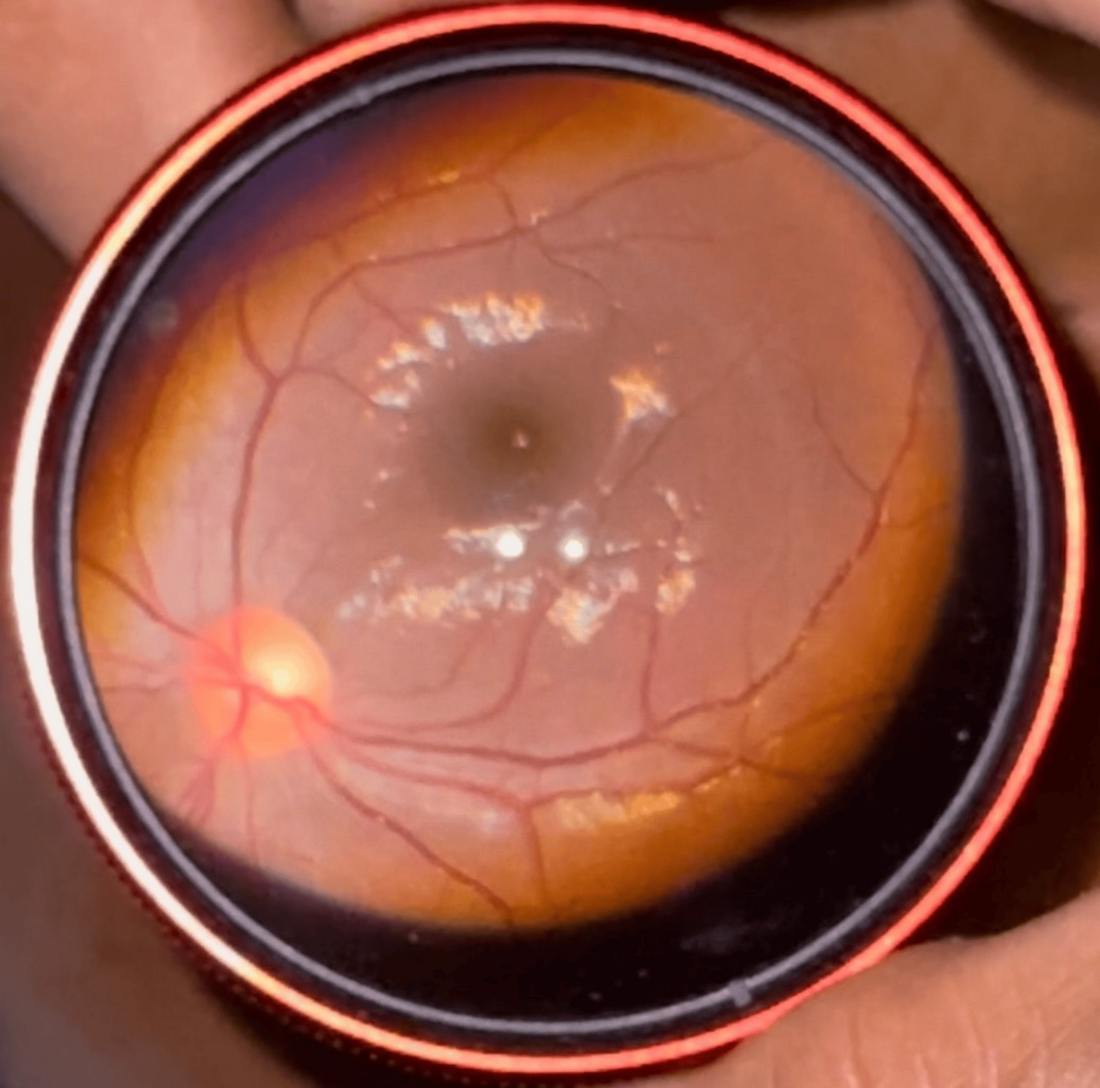
Right eye fundus showing normally appearing optic disc and retinal vessels with the presence of macular foveal reflex

**Figure 3:**
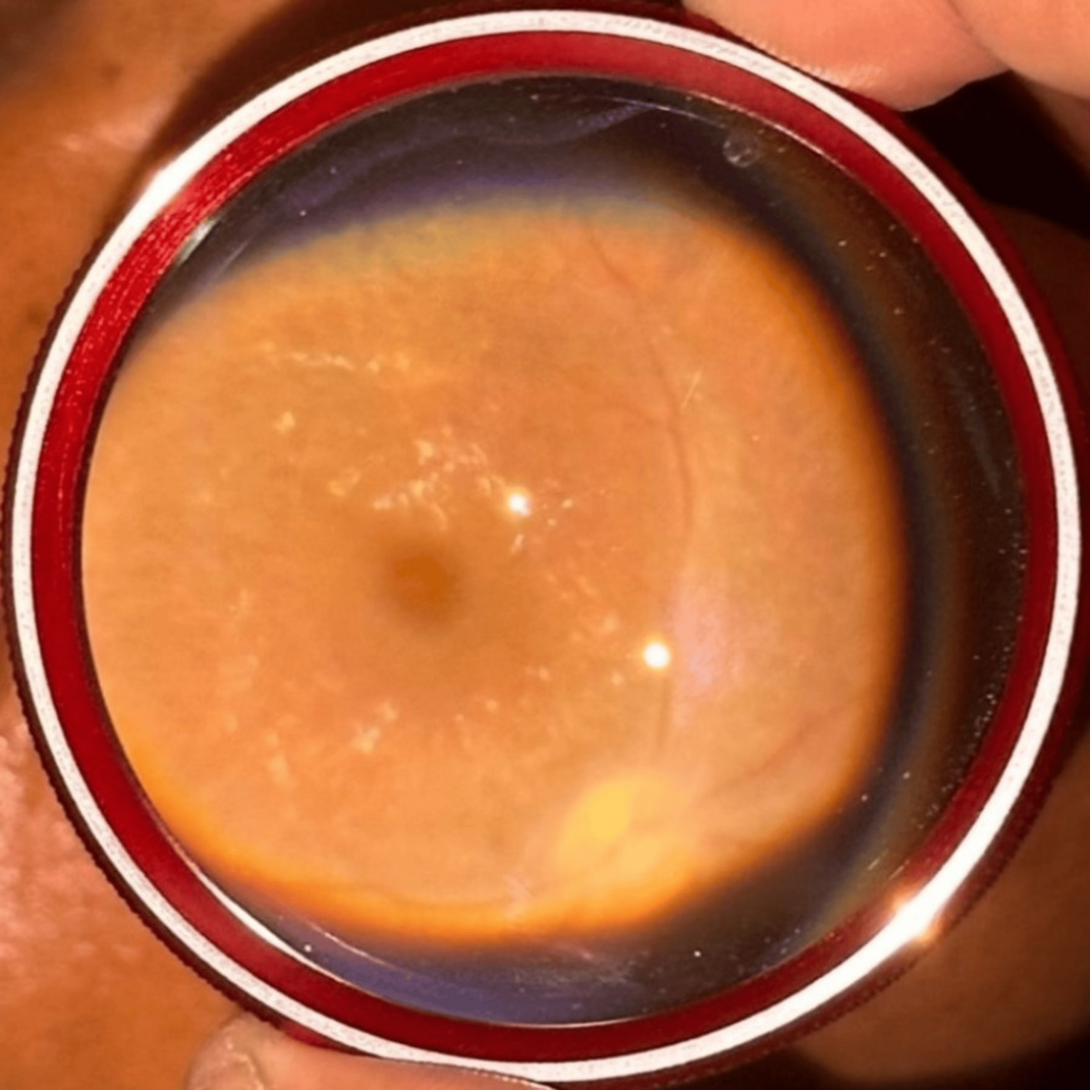
Fundus showing pale retina, disc pallor, attenuated retinal vessels, and an absent cherry red spot in the left eye

The dilated fundus examination was done at the bedside with an indirect ophthalmoscope using a 20-dpt lens. The disc and vessels appeared normal, with the presence of a macular foveal reflex in the right eye, as can be seen in Figure 2. The left eye was noted to have a pale retina, optic disc pallor, severe retinal vessel attenuation, and an absence of a cherry red spot, as can be seen in Figure 3. These clinical features are typical of ophthalmic artery occlusion.

**Figure 4:**
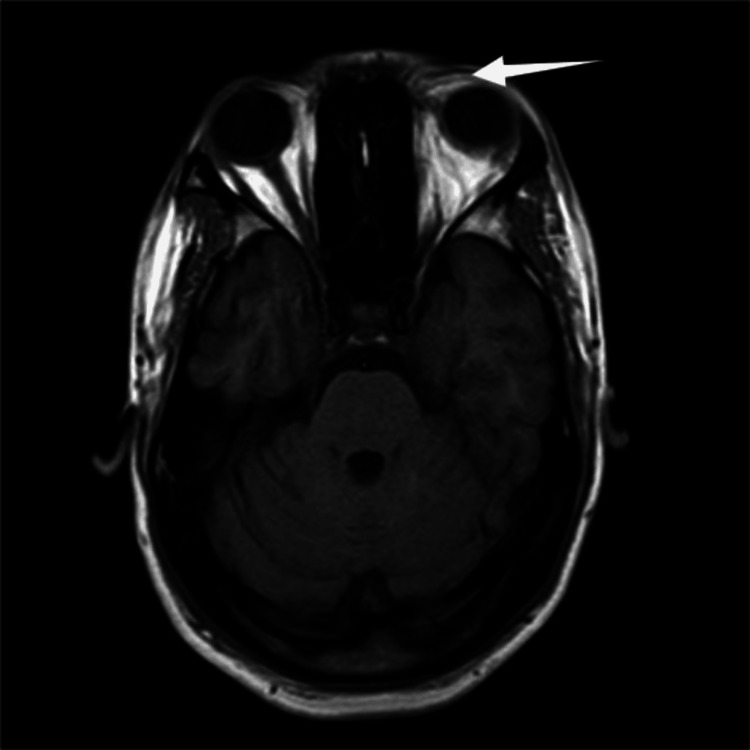
MRI T2 FLAIR axial image of the orbit, showing mild soft-tissue hyperintensities (white arrow) in the medial aspect of the preseptal region of the left orbit, suggestive of preseptal edema MRI: magnetic resonance imaging; FLAIR: fluid-attenuated inversion recovery

The magnetic resonance imaging report of the patient's orbit revealed mild soft-tissue hyperintensities (white arrow) in the medial aspect of the preseptal region of the left orbit, suggestive of preseptal edema, as seen in Figure 4. Magnetic resonance venography reports were normal. Echocardiogram results were also normal. However, further investigations such as optical coherence tomography (OCT), electroretinogram, and fundus fluorescein angiography were not conducted due to the patient's nonambulatory status.

**Figure 5:**
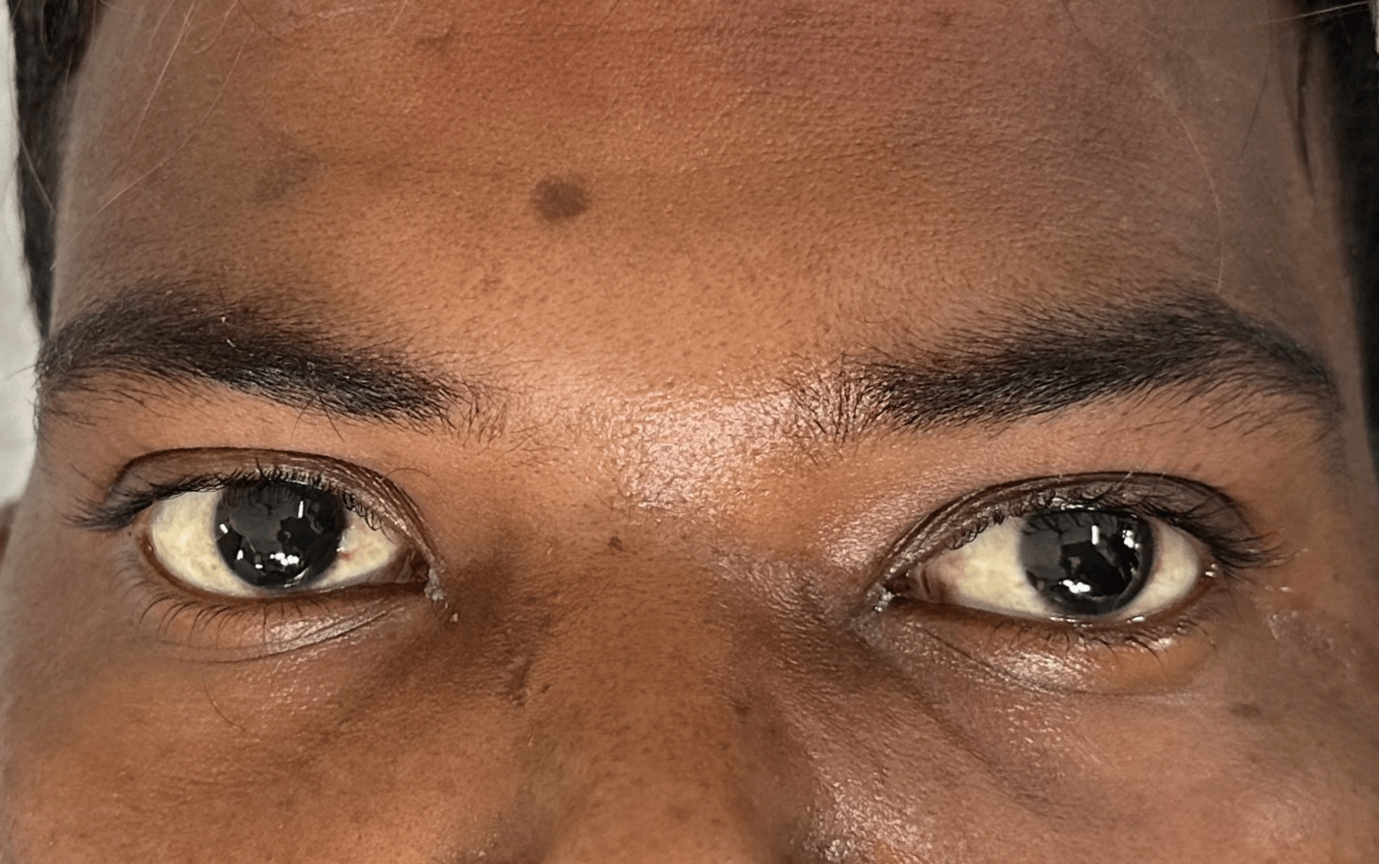
Resolved supraorbital edema, conjunctival congestion, and chemosis in the left eye

The patient was started on IV methylprednisolone 1 g for three days and then tapered to oral steroids. Daily visual monitoring and anterior segment examination were done at the bedside. On the fifth follow-up day, the congestion, chemosis, and supraorbital edema resolved in the left eye, as seen in Figure 5. On the 10th follow-up day, the restriction of eye movements recovered with only mild restriction on abduction in the left eye, but the vision in the left eye was still the only perception of light. The patient was bedridden until a one-month follow-up due to a grade 3 sacral pressure sore, for which wound debridement was done. On the one-month follow-up, the vision did not improve in the left eye, but there was no restriction in extraocular movements noted with a similar fundus picture as before in the left eye. The patient was discharged at request due to financial burden and lost to follow-up.

## Discussion

Though many causes of POVL exist, including CRAO, ION, and cortical blindness, their exact etiologies are still unclear [9,10]. This may be partly because of the lack of clarity about the pathoanatomy of POVL, given the intricate and highly variable nature of the ocular vasculature [11].

The following characteristics should be noted when diagnosing a case based on clinical findings alone. A pale ischemic retina with a pathognomonic cherry red spot in the macula and a relative afferent pupillary deficit or impaired pupillary light reflex is required in the case of a CRAO. An early fundus examination showing optic disc edema with or without peripapillary flame-shaped hemorrhages and a relative afferent pupillary defect or impaired pupillary light reflex should be demonstrated in anterior ION. Criteria consistent with posterior ischemic optic neuropathy included a normal early funduscopic examination with a relative afferent pupillary defect or an absent pupillary light reflex [12].

Occlusions of the ophthalmic arteries are typically found close to the central retinal artery that supplies the retina and the posterior ciliary arteries that supply the choroid. Thus, acute occlusion of the retinal and choroidal circulations due to blockage of the ophthalmic artery causes significant visual impairment. A cherry red spot may not be seen on funduscopic examination due to ischemia in the choroid and retinal circulations with insufficient blood flow to the entire retina, including the foveal region [7].

In our case report, the patient presented with sudden painless loss of vision in the left eye postoperatively with typical clinical features of ophthalmic artery occlusion showing pale retina, marked optic disc pallor with retinal arteriole as well as venules attenuation, and absent cherry red spot in fundus examination owing to ischemic choroid and retinal vessels. The local ocular signs could have resulted from direct pressure on the globe and the periorbital structures during an extended prone position, as stated by Hayreh [13]. The ophthalmoplegia could be explained by the partial or complete collapse of the arterial and venous channels of the orbit due to the tamponade action of the ocular contents secondary to the compression with the reversibility of the ophthalmoplegia depending on the severity of damage to the extraocular muscles as well as the cranial nerves [1]. There were no additional preoperative factors that might have played a role in his vision loss, as he was a nonsmoker and nontobacco user with no known systemic illnesses.

There are many case reports in the literature about postoperative acute unilateral visual loss following nonocular surgeries, with the noted cause being CRAO or ION. But visual loss associated with ophthalmoplegia is reported less with few in literature, including case reports by Mukherjee and Alam [14] and Raj et al. [15], in which CRAO was attributed as the cause for visual loss and complete ophthalmoplegia following an uneventful spinal surgery.

Case reports on ophthalmic artery occlusion following spine surgery are even rarer, with a notable case report by Kothari and Maiti where a nine-year-old child underwent uneventful spinal surgery lasting 6.6 hours in a prone position with 150-200 mL blood loss and a hemoglobin level of 12.5 g/dL complained of complete loss of vision in the left eye after surgery prompting ophthalmology evaluation [16]. Examination revealed no light perception in the left eye, with characteristic findings indicating ophthalmic artery occlusion, which was confirmed by typical features and OCT. The cause was attributed to prolonged compression during surgery, a known risk factor. It was also stated that visual loss after surgery can result from factors like blood loss, anemia, hypotension, or increased intracranial pressure, emphasizing the need for vigilant monitoring and prevention strategies.

Kumar et al., in the case report of visual loss after spine surgery, reported that spinal surgery is the leading cause of POVL and that the horseshoe is no longer a useful tool for prone spinal surgery, and its application ought to be discouraged [17].

Similarly, Sperber et al. conclude that vision loss following nonocular surgery, especially spinal surgery, is a devastating complication that affects recovery from surgery as well as the quality of life and that the prevalence of POVL will rise in proportion to the sharp rise in spine surgery rates [18].

Hence, understanding POVL's pathophysiological mechanisms and clinical presentations is crucial [19]. This will aid in early recognition, prompt intervention, and multidisciplinary collaboration, thus mitigating the impact of this alarming complication [20].

## Conclusions

Patients undergoing prone surgery of a long duration are at the greatest risk of experiencing POVL. A preoperative discussion and informed consent regarding postoperative visual loss should be done for every patient undergoing surgery in the prone position. The general outcome of postoperative visual loss is often poor due to the absence of effective treatment options and unclear pathogenesis. Future studies are required to develop effective treatments. Hence, at present, both the anesthetist and surgeon must be mindful of the positioning of the patient, the duration of the surgery, maintenance of vitals within normal range throughout surgery, and refraining from applying any external ocular compression, especially the use of horseshoe headrest so as to avert this complication. It is also essential to closely monitor intraoperative blood loss as it can also contribute to POVL.
